# Serum markers of B‐cell activation in pregnant women with atopic asthma

**DOI:** 10.1111/aji.13414

**Published:** 2021-03-19

**Authors:** Catarina Martins, Jorge Lima, Geraldine Cambridge, Miguel Ângelo‐Dias, Maria Leandro, Luís Miguel Borrego

**Affiliations:** ^1^ CEDOC NOVA Medical School Nova University of Lisbon. Campo dos Mártires da Pátria Lisbon Portugal; ^2^ Comprehensive Health Research Centre (CHRC) NOVA Medical School Nova University of Lisbon Lisbon Portugal; ^3^ Department of Obstetrics and Gynecology CUF Descobertas Hospital Lisbon Portugal; ^4^ Centre for Rheumatology and Bloomsbury Rheumatology Unit Division of Medicine University College London London UK; ^5^ Department of Imunoallergy LUZ SAÚDE, Hospital da Luz Lisbon Portugal

**Keywords:** allergy, asthma, B‐cell activating factor, free light chain, immunoglobulins, postpartum, pregnancy, soluble CD23

## Abstract

**Problem:**

As maternal atopy represents a risk factor for the development of atopy in offspring, we aimed to assess how pregnancy affects B‐cell activation markers in women with atopic asthma and whether they correlate with risk manifestations for allergy in newborns from mothers with atopic asthma.

**Method of Study:**

Pregnant women with atopic asthma (AP) in the third trimester of gestation and nonpregnant women with atopic asthma (ANP) were prospectively recruited and compared to respective healthy counterparts (HP and HNP). All pregnant women were also assessed during the postpartum period until 6 weeks after delivery (HP/PP and AP/PP). Newborns were clinically evaluated at the age of 6 months. Peripheral blood samples were taken from each woman at each time point. Soluble CD23 (sCD23), B‐cell activating factor (BAFF), IgA, IgG, IgM, kappa (κ), and lambda (λ) free light chains (FLC) were quantified in serum samples.

**Results:**

The AP group presented increased sCD23 (*p* < 0.05) and BAFF (*p* < 0.001) levels compared to the ANP group and even higher levels of sCD23 during the postpartum period (*p* < 0.001). Moreover, the cutoffs of 6.74 g/L for IgG (sensitivity 90.9%, specificity 77.8%) and of 11.30 mg/L for *λ* FLC (sensitivity 81.8%, specificity 88.9%) in the AP group were predictive factors for the manifestation of allergy in their offspring.

**Conclusions:**

After delivery, the dynamics of sCD23 and BAFF changed significantly in the AP group. Furthermore, we found novel predictive factors for allergy manifestations in the children of these women, with potential clinical application.

## 1 INTRODUCTION

A successful pregnancy outcome strongly depends on immune modulation during gestation, which involves highly regulated mechanisms that ensure tolerance induction for the genetical foreign content while maintaining necessary protection of the both mother and developing fetus.^1^, ^2^

Atopic diseases, such as allergic asthma and allergic rhinitis, are some of the most common chronic disorders in pregnant women.^3^, ^4^ Women with these conditions have several risk factors and an increased probability of adverse pregnancy outcomes.^5^, ^6^ Moreover, reports have shown that the course of atopic diseases such as asthma may worsen, improve, or remain unchanged during pregnancy.^7^, ^8^ However, the factors underlying these manifestations remain to be elucidated.

Recently, the role of B cells in maintaining maternal‐fetal tolerance and the protective equilibrium state observed during pregnancy has been widely studied.^9^, ^10^, ^11^ In fact, despite their classic humoral functions, B cells are also involved in regulatory activities.^1^ Several studies have reported interesting modulations of B cells during pregnancy, such as B‐cell lymphopenia, and an impairment in more differentiated B‐cell subsets.^12^ Indeed, the circulating B‐cell compartment undergoes significant quantitative and qualitative changes from the third trimester of pregnancy to the postpartum period.^12^, ^13^, ^14^, ^15^ Moreover, obstetric complications have been associated with the production of autoantibodies, which underlie abnormal B‐cell function.^16^, ^17^, ^18^ It is thus relevant to better understand how B‐cell‐related immune players behave during the gestational and postpartum periods.

CD23, also known as Fc epsilon RII, is a low‐affinity IgE receptor found on the surface of naïve B cells. After B‐cell activation, expression of CD27 induces cleavage of CD23 to its soluble form (sCD23). Thus, levels of CD23 can be used to assess the turnover of B cells from a naïve to a memory state.^19^, ^20^, ^21^ B‐cell activating factor (BAFF) is a fundamental survival factor for B cells, and it is particularly important in the differentiation of immature transitional B cells into mature naïve B cells.^22^ Recently, we reported that variations in circulating B‐cell subsets during pregnancy are associated not only with changes in the serum levels of these B‐cell function markers but also in the serum concentrations of immunoglobulin (Ig) and free light chains (FLC). According to our previous results, late pregnancy is accompanied by a significant increase in BAFF levels and a decrease in sCD23 levels, followed by an increase in the levels of both markers during the postpartum period.^23^ These data suggest that B‐cell activation occurs during pregnancy but that there may be limitations in the normal differentiation of memory subsets and, consequently, lower amounts of CD23 shed.^23^

The involvement of B cells in allergic responses through the production of allergen‐specific Ig has been widely studied. We previously reported that nonpregnant women with atopic diseases have a distinctive B‐cell compartment that becomes masked with pregnancy.^24^ In fact, the maturation profile of B cells is quite similar in healthy pregnant women and those with atopic disease, though differences in transitional B cells are found.^13^ These similarities have also been described for the CD24^Hi^CD38^Hi^ transitional B cell subset, which is enriched in regulatory B cells.^24^ However, data regarding the effects of pregnancy on the circulating B‐cell compartment of women with atopic disease and vice versa are still scarce. In addition, we found that transitional B cells help predict allergic manifestations in the progeny of women with atopic disease.^13^ Nevertheless, the predictive factors for allergy manifestations in their children are still largely unknown.

In this study, we aimed to characterize serum sCD23, BAFF, IgG, IgM, IgA, and kappa (κ) and lambda (λ) FLC in women with atopic asthma, to assess their variations during late pregnancy and the postpartum period and to compare these variations between healthy pregnant and nonpregnant women. We also aimed to determine whether these markers are predictive factors for the identification of allergy manifestations in the progeny of women with atopic asthma.

## 2 MATERIALS AND METHODS

### 2.1 Study design and participants

This was a prospective observational study that included two cohorts of women of childbearing potential: one cohort of healthy nonpregnant (HNP) or pregnant (HP) women and one cohort of nonpregnant (ANP) or pregnant (AP) women with atopic asthma. Both cohorts were recruited and followed‐up at CUF Descobertas Hospital (Lisbon, Portugal) between July 2013 and March 2014. These cohorts were described in previous studies.^12^, ^13^, ^23^, ^24^ Pregnant women from the third trimester of pregnancy (31st‐36th weeks of gestation) were included and were followed‐up until 6 weeks after delivery. At the age of 6 months, all babies were assessed for potential allergy risk manifestations. All participants signed a written informed consent prior to their inclusion in the respective study. The study protocols were approved by the Hospital and NOVA Medical School Ethics Committees and were conducted according to the Declaration of Helsinki.

The women were divided into the following groups according to eligibility criteria: HNP and HP further assessed as healthy postpartum women (HP/PP); and ANP and AP further assessed as postpartum women with atopic asthma (AP/PP).

For all groups, only women of childbearing potential were included. The exclusion criteria were as follows: pathologies such as diabetes, hypertension, autoimmune, or any active infectious disease including hepatitis and human immunodeficiency virus (HIV), any other allergic or respiratory disease (except atopic asthma), and smoking in the previous 6 months before sample collection.^12^, ^13^, ^23^, ^24^ Accordingly, the healthy groups were defined as not having any of the abovementioned features or atopic asthma.

The groups with atopic asthma were defined as having asthma with rhinitis (diagnosed according to international guidelines^25^, ^26^), with proven atopy. Only patients with controlled asthma (according to the Global Initiative for Asthma (GINA) guidelines^25^) were included.^13^, ^24^ Proven atopy was considered when the presence of sensitization to aeroallergens had been documented by skin prick tests and/or specific IgE quantification. The women of the ANP and AP groups were under anti‐inflammatory therapy (low median dose of inhaled corticosteroid 200–400 μg beclomethasone/daily and/or long‐acting beta‐agonists and antileukotrienes). Women with asthma exacerbation in the 6 weeks preceding the blood sample collection were excluded from the study.

For the pregnancy groups, only pregnant women with uncomplicated and singleton pregnancies were included.^12^, ^13^, ^23^, ^24^ The exclusion criteria of both the healthy pregnant women and those with atopic asthma were the use of any prenatal medication other than vitamins, folic acid, and iron supplements, with the exception of treatments for atopic disease in the AP group. All pregnant women were enrolled during the third trimester (between gestational weeks 31 and 36) to better exclude the occurrence of complications, such as asthma exacerbations, which are more prone to occur in the second trimester. Postpartum was defined as the period until 6 weeks after delivery. The presence of eczema, recurrent wheeze (≥3 episodes in the first 6 months of age), and food allergy were evaluated in newborns at 6 months of age. According to the presence or absence of these allergic manifestations, the AP group was further divided into AP with allergic manifestations in progeny (AMP) and AP without allergic manifestations in progeny (NAMP).

### 2.2 Immunophenotypic characterization of lymphocyte B‐cell subsets

Peripheral blood samples were collected into EDTA‐coated and heparinized tubes and analyzed in a four‐color BD FACSCalibur flow cytometer (BD Biosciences). Briefly, the enumeration of B cells was performed using the BD Multitest IMK kit (BD Biosciences), and B‐cell subsets were further characterized, using the following surface markers: CD19, CD24, CD27, CD38, IgD, and anti‐IgM. Subset analysis was performed as previously described and included the assessment of Breg related subsets, such as memory CD24^Hi^CD27^+^ B cells and transitional CD24^Hi^CD38^Hi^ B‐cells.^12^, ^13^, ^27^, ^28^ Moreover, IL‐10 secreting B‐cells were quantified in peripheral blood, after a 5 hours stimulation with PMA (50 ng/mL), calcium ionophore (1 μg/mL), and LPS (10 μg/mL), at 37°C in a 5% CO_2_‐enriched atmosphere, using the intracellular immunophenotyping protocol described elsewhere.^13^ All gating strategies are displayed in Figure S1.

### 2.3 B‐cell activation markers in serum samples

Serum was obtained after coagulum retraction and sample centrifugation. Aliquots were then separated, stored at −20°C, and sent to University College London for further quantification of circulating sCD23, BAFF, and Ig levels.

Commercial enzyme‐linked immunosorbent assay (ELISA) kits were used for the assessment of both serum sCD23 and BAFF levels. For sCD23 quantification, serum samples were analyzed with Human Quantikine® sCD23 (R&D Systems Europe Ltd). The normal range of sCD23 was defined according to the manufacturer (1235–5024 pg/mL). For BAFF quantification, serum samples were analyzed with the Human Quantikine^®^ BAFF/BLyS immunoassay ELISA kit (R&D Systems Europe Ltd). Normal BAFF ranges were defined by the laboratory cohort (200–1186 pg/mL).

Total IgA, IgG, and IgM serum concentrations (mg/L) were determined using in‐house nephelometry by Binding Site Group Ltd. Normal ranges were defined as 0.845–4.99 g/L for IgA, 6.103–16.16 g/L for IgG, and 0.35–2.42 g/L for IgM.

In addition, κ FLC and λ FLC serum concentrations (mg/L) were evaluated using ELISA commercial kits (Biovendor) according to the manufacturer's instructions. Normal ranges were defined as 3.3–19.4 mg/L for κ FLC, 5.71–26.3 mg/L for λ FLC, and 0.20–1.65 for κ/λ FLC ratio.

### 2.4 Statistical analysis

Continuous normally distributed data are expressed as the mean and standard deviation (SD); non‐normally distributed data are expressed as the median and interquartile range [IQR]. Normality of distributions was assessed using the D'Agostino and Pearson test.

For normally distributed data, comparisons between two independent groups were performed using the unpaired *t*‐test with Welch's correction; otherwise, the nonparametric Mann–Whitney *U*‐test was applied. Comparison between three or more independent groups was performed with Kruskall‐Wallis test. Paired data were compared using paired Student's *t* test or the Wilcoxon signed‐rank test, as appropriate.

Spearman's rank correlation test was performed to analyze correlations between B‐cell subsets and levels of B‐cell activation markers.

To assess the ability of B‐cell activation markers to discriminate between women of the AP group with and without atopic manifestations in offspring, receiver operating characteristic (ROC) curves and areas under the curve (AUCs) were examined, followed by the application of Fisher's exact test to contingency tables. The cutoffs of B‐cell activation markers were determined through ROC analysis based on the best balance between sensitivity and specificity, with 95% confidence intervals (CI). The association between immune mediators and atopic manifestations in the children of women of the AP group was further assessed by logistic regression analysis. These results are expressed as odds ratios (ORs) with 95% CIs.

Statistical significance was considered at *p* < 0.05. All analyses were performed using GraphPad Prism version 8.4.2 (GraphPad), with the exception of logistic regression analysis, which was performed using the R package (version 3.5.3).

## 3 RESULTS

### 3.1 Study population

Demographic and anthropometric data of all women included in the study are summarized in Table 1. This study included a total of 130 participants: 35 HNP, 43 HP, 32 ANP, and 20 AP. The median age was 35 years for HNP, 32 years for HP, 36 years for ANP, and 34 years for AP, with no significant differences between groups with regard to age, ethnicity, or education level. Only body mass index (BMI) was significantly increased in pregnant women compared to both the HNP and ANP groups. For the two pregnancy groups, no differences were identified for parity, smoke exposure, gestational age at evaluation and at parturition, time of postpartum assessment, or sex of the baby.^24^

**TABLE 1:** Baseline characteristics of nonpregnant and pregnant women with and without asthma and their newborns

Characteristics	HNP (*n* = 35)	HP (*n* = 43)	ANP (*n* = 32)	AP (*n* = 20)	*p*‐value
Age, median [IQR], years	35 [4]	32 [5]	36 [6]	33 [4]	0.071^a^
BMI, median [IQR], kg/m^2^	21.1 [2.4]	25.6 [4.1]	21.9 [5.0]	25.9 [4.5]	**<0.001** ^a^
Gestational age (complete weeks) at inclusion, median [IQR]		33 [2]		34 [1]	0.081^b^
Gestational age (complete weeks) at delivery day, median [IQR]		38 [1]		39 [2]	0.489^b^
Type of delivery, No. (%)					
Vaginal		18 (41.9)		13 (65.0)	0.109^c^
Cesarean		25 (58.1)		7 (35.0)	
Newborns birth weight, mean (SD), g		3199 (402)		3282 (352)	0.411^d^

Note

Statistically significant results are indicated in bold.

Abbreviations: ANP, atopic asthmatic nonpregnant women; AP, atopic asthmatic pregnant women; BMI, body mass index; HNP, healthy nonpregnant women; HP, healthy pregnant women; IQR, interquartile range; SD, standard deviation.

^a^ Kruskal‐Wallis test

^b^ Mann‐Whitney nonparametric *U*‐test

^c^ Fisher's exact test

^d^ Unpaired Student's *t* test.

Overall, 45% of women in the AP group had children with allergic manifestations.

### 3.2 Serum sCD23 levels in healthy women and those with atopic asthma

**FIGURE 1:**
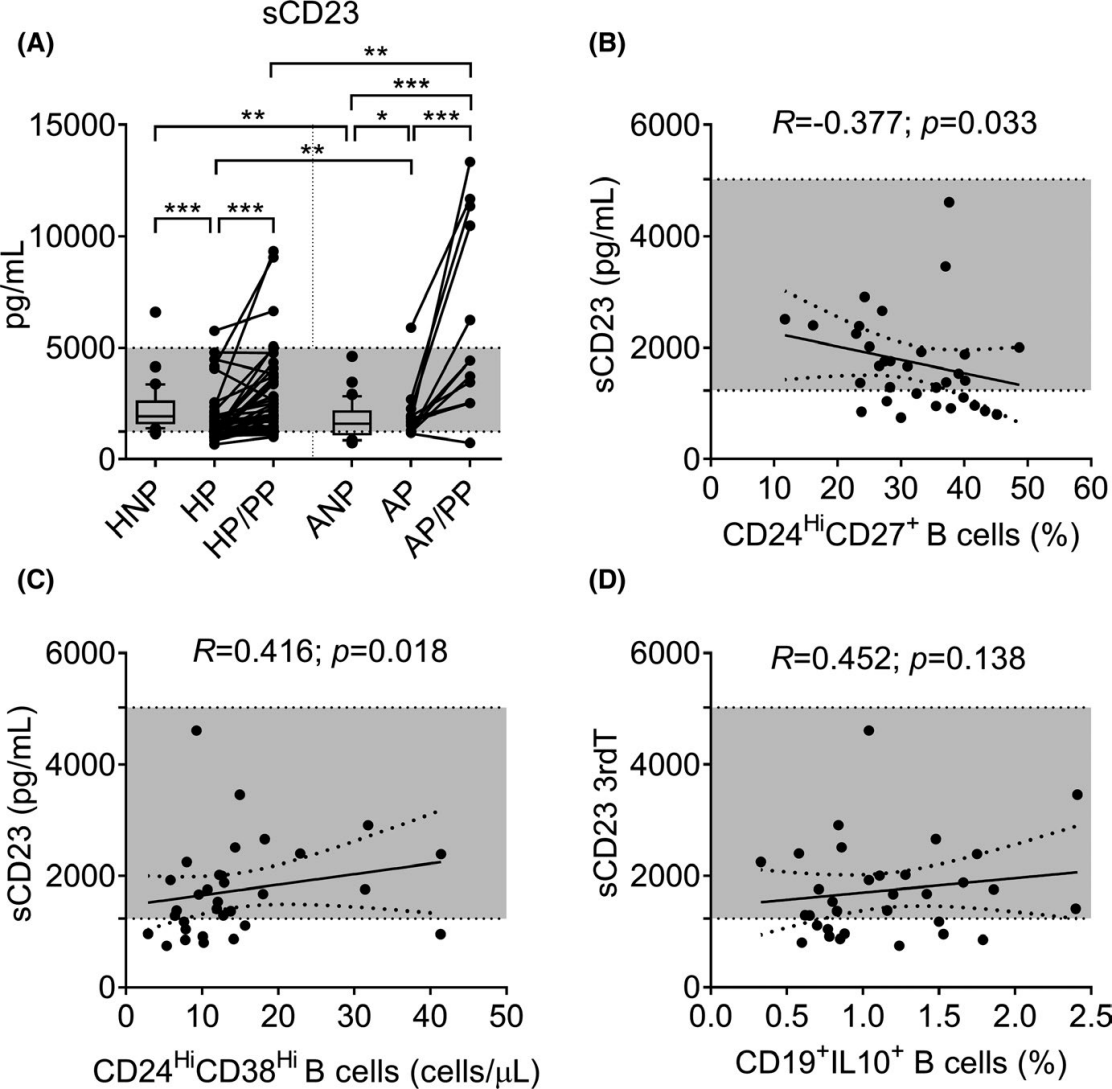
Box (median with IQR) and whisker (10th and 90th percentiles), and before‐after plots of sCD23 serum levels in the peripheral blood of healthy nonpregnant women and those with atopic asthma (HNP and ANP), during the third trimester of pregnancy (HP and AP), and during the postpartum period at least 6 weeks after delivery (HP/PP and AP/PP). Differences between unpaired and paired groups were tested using the Mann‐Whitney *U*‐test or Wilcoxon signed‐rank test, respectively (A). Correlations between sCD23 serum levels and CD24^Hi^CD27^+^ B‐cells (B), CD24^Hi^CD38^Hi^ B‐cells (C) and IL‐10 secreting B‐cells (D) in ANP women. For (B), (C) and (D), Spearman correlation coefficients, 95% confidence intervals, and *p*‐values are indicated. HNP: *n* = 35; HP: *n* = 43; HP/PP: *n* = 42; ANP: *n* = 32; AP: *n* = 20; AP/PP: *n* = 12. *p‐value <0.05; **p‐value <0.01; ***p‐value <0.001. Shaded areas represent normal range. All data regarding healthy women was adapted from Lima et al.^23^

The serum levels of sCD23 for all the studied groups are presented in Figure 1A. We found differences in the levels of sCD23 between the groups. Specifically, sCD23 levels were significantly increased in the HNP group compared to the ANP group (*p* = 0.010). Within the healthy groups, we have previously reported lower levels of sCD23 in HP compared to HNP group (*p* < 0.001). In contrast, the AP group showed significantly higher levels of sCD23 compared to both the ANP (*p* = 0.039) and HP (*p* = 0.004) groups (Figure 1A).

During the postpartum period, both healthy and those with atopic asthma presented higher levels of sCD23 than those found during the third trimester of pregnancy (*p* ≤ 0.001). Interestingly, although sCD23 levels of women in the HP/PP group were similar to those observed in the HNP group (*p* = 0.730), sCD23 levels in the AP/PP group were significantly increased compared to the ANP group (*p* < 0.001). Moreover, when comparing both groups of postpartum women, the levels of sCD23 were higher in those with atopy than in healthy women (*p* = 0.002; Figure 1A).

We also found that the levels of sCD23 in the ANP group correlated negatively with the percentages of the CD24^Hi^CD27^+^ B‐cells subset (Figure 1B; *R* = −0.377, *p* = 0.033) and positively with the absolute counts of the CD24^Hi^CD38^Hi^B‐cells subset (Figure 1C; *R* = 0.416, *p* = 0.018). No further correlations were detected between sCD23 and regulatory or IL‐10‐secreting B cells during pregnancy or the postpartum period of the women with atopic asthma.

Regarding the healthy groups, a tendency for a positive correlation between sCD23 and the absolute counts of the CD24^+^CD38^+^ B‐cells subset (data not shown, *R* = 0.329, *p* = 0.054) was only observed in the HNP group.

## 4 SERUM BAFF lEVELS IN HEALTHY WOMEN AND THOSE WITH ATOPIC ASTHMA

**FIGURE 2:**
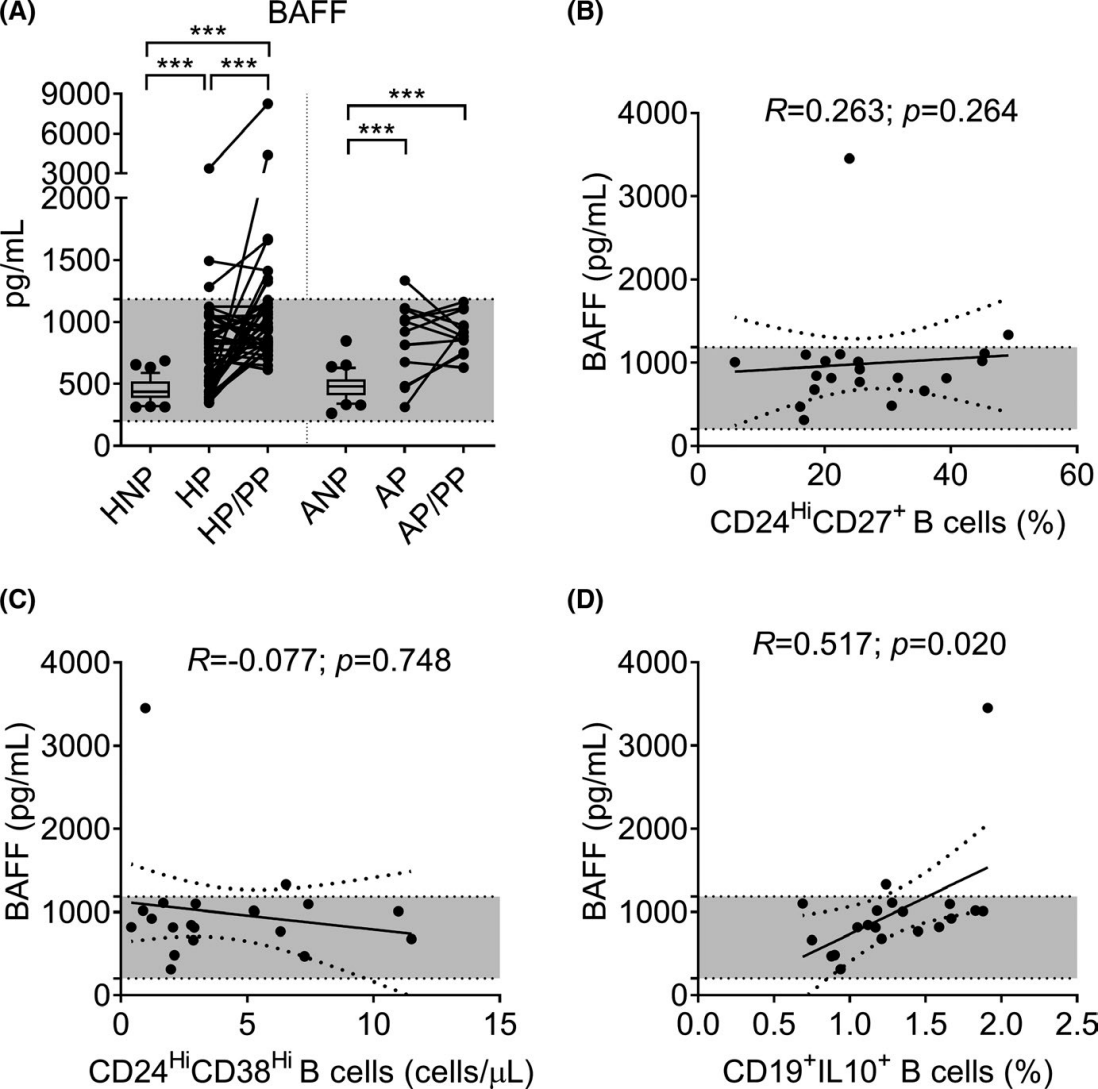
Box (median with IQR) and whisker (10th and 90th percentiles), and before‐after plots of BAFF serum levels in the peripheral blood of healthy nonpregnant women and those with atopic asthma (HNP and ANP), during the third trimester of pregnancy (HP and AP), and during the postpartum period at least 6 weeks after delivery (HP/PP and AP/PP). Differences between unpaired and paired groups were tested using the Mann‐Whitney *U*‐test or Wilcoxon signed‐rank test, respectively (A). Correlation between BAFF serum levels and CD24^Hi^CD27^+^ B‐cells (B), CD24^Hi^CD38^Hi^ B‐cells (C) and IL‐10 secreting B‐cells (D) in AP women. For (B), (C) and (D), Spearman correlation coefficients, 95% confidence intervals, and P‐values are indicated. HNP: *n* = 35; HP: *n* = 43; HP/PP: *n* = 43; ANP: *n* = 32; AP: *n* = 20; AP/PP: *n* = 12. *p‐value <0.05; **p‐value <0.01; ***p‐value <0.001. Shaded areas represent normal range. All data regarding healthy women were adapted from Lima et al.^23^

The serum BAFF levels for all the studied groups are depicted in Figure 2A.

Similar to what we have found in HP women, significantly increased serum levels of BAFF were observed in both the HP and AP groups compared to nonpregnant counterparts (Figure 2A; *p* < 0.001). However, no differences between the pregnancy (AP vs. HP) and nonpregnancy groups (ANP vs. HNP) were observed.

Interestingly, only healthy women showed significantly increased median serum BAFF levels during the postpartum period compared to the third trimester of pregnancy (Figure 2A; *p* = 0.001). In addition, serum BAFF levels were significantly elevated during the postpartum period in both healthy women and those with atopic asthma compared to their nonpregnant counterparts (*p* < 0.001).

Finally, within the atopic group, pregnant women showed a positive correlation between serum BAFF levels and IL‐10‐secreting B‐cell percentages in the third trimester of pregnancy (Figure 2D; *R* = 0.517, *p* = 0.020). No further differences or correlations were identified for this marker.

## 5 SERUM LEVELS OF IMMUNOGLOBULINS IN HEALTHY WOMEN AND THOSE WITH ATOPIC ASTHMA

**FIGURE 3:**
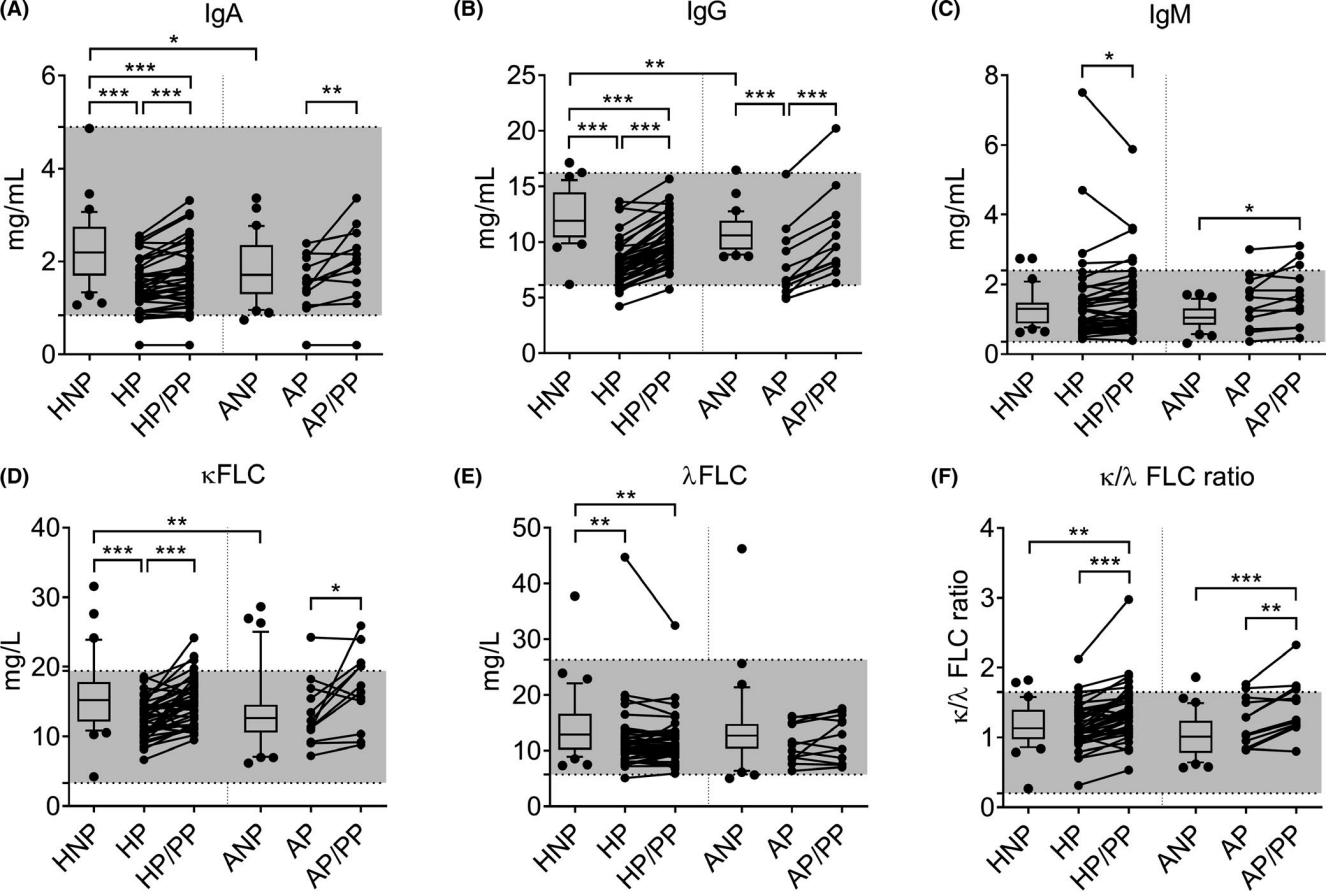
Box (median with IQR) and whisker (10th and 90th percentiles), and before‐after plots of total serum levels of (A) IgA, (B) IgG, (C) IgM, (D) κ FLC, (E) λ FLC, and (F) k/λ FLC ratio in the peripheral blood of healthy nonpregnant women and those with atopic asthma (HNP and ANP), during the third trimester of pregnancy (HP and AP), and during the postpartum period at least 6 weeks after delivery (HP/PP and AP/PP). Differences between unpaired and paired groups were tested using the Mann‐Whitney *U*‐test or Wilcoxon signed‐rank test, respectively. HNP: *n* = 35; HP: *n* = 43 (*n* = 42 in IgA); HP/PP: *n* = 43 (*n* = 42 in IgA); ANP: *n* = 32; AP: *n* = 20; AP/PP: *n* = 12. *p‐value <0.05; **p‐value <0.01; ***p‐value <0.001. Shaded areas represent normal range. All data regarding healthy women was adapted from Lima et al.^23^

The serum levels of immunoglobulins for all the studied groups are illustrated in Figure 3A–C.

Levels of IgA were significantly higher in the HNP group than in both the ANP (*p* = 0.018) and HP (*p* < 0.001) groups. In addition, IgA increased in the postpartum period compared to the third trimester of pregnancy in healthy women and in those with atopic asthma (*p* ≤ 0.007). Interestingly, the HP/PP group presented significantly lower IgA levels than the HNP group (*p* = 0.001), though this difference was not detected when comparing the ANP and AP/PP groups (*p* = 0.510; Figure 3A).

Levels of IgG were significantly decreased in the HP and AP groups compared to the HNP and ANP groups, respectively (*p* < 0.001). Furthermore, HNP was associated with higher IgG levels than ANP (*p* = 0.006; Figure 3B). As observed for IgA levels, IgG levels increased significantly in the postpartum period compared to the third trimester of pregnancy in both healthy women and in those with atopic asthma (*p* ≤ 0.001). Again, only healthy women showed significantly decreased IgG levels during the postpartum period compared to their nonpregnant counterparts (*p* < 0.001; Figure 3B).

As previously described, levels of IgM were significantly increased in the HP/PP group (*p* = 0.028) compared to the HP group. Regarding women with atopic asthma, AP/PP showed higher IgM levels compared to their respective nonpregnant counterparts (*p* = 0.021), as well as a tendency for higher levels compared to AP (*p* = 0.064). No further significant differences in median IgM levels were found between groups, though a tendency for lower levels in ANP compared to HNP (*p* = 0.055) was observed (Figure 3C).

## 6 SERUM LEVELS OF κ AND λ FCL IN HEALTHY WOMEN AND THOSE WITH ATOPIC ASTHMA

The serum levels of κ and λ FLC for all the studied groups are presented in Figure 3D–F.

Overall, serum κ and λ FLC levels followed the same pattern observed for IgA and IgG levels, respectively (Figure 3D,E). Specifically, we observed that κ FLC levels were significantly increased in HNP compared to both ANP (*p* = 0.008) and HP (*p* < 0.001). Additionally, levels of κ FLC were significantly higher during the postpartum period than during the third trimester of pregnancy in both the healthy women (*p* < 0.001) and in those with atopic asthma (*p* = 0.043). Interestingly, although levels of κ FLC were similar between HNP and HP/PP, κ FLC levels exhibited a tendency to be higher in the postpartum period of women with atopic asthma compared to their nonpregnant counterparts (*p* = 0.052; (Figure 3D). Regarding λ FLC, higher levels were found for HNP compared to both HP and HP/PP (*p* ≤ 0.009), without differences in the women with atopic asthma (Figure 3E). These alterations were reflected by the significant differences observed in the κ/λ FLC ratio (Figure 3F).

### 6.1 Predictive factors for allergy manifestation in the children of women with atopic asthma

The comparison of B‐cell activation markers in AP group with (AP AMP) and without (AP NAMP) allergy manifestations in their progeny is provided in Table 2.

**TABLE 2:** Comparison of B‐cell activation markers in AP AMP and AP NAMP groups

B‐cell activation markers	AP AMP^a^ (*n* = 9)	AP NAMP^a^ (*n* = 11)	*p*‐value^b^
sCD23 (pg/mL)	1827 [1107]	2507 [1146]	0.112
BAFF (pg/mL)	1006 [439]	818 [344]	0.824
IgA (mg/mL)	1.53 [0.58]	1.64 [1.02]	0.710
IgG (mg/mL)	6.40 [2.02]	8.33 [2.20]	**0.031**
IgM (mg/mL)	0.93 [0.59]	0.93 [1.50]	0.656
κ FLC (mg/L)	10.95 [2.86]	13.45 [3.72]	0.056
λ FLC (mg/L)	8.77 [3.17]	12.10 [4.27]	**0.001**
κ/λ FLC ratio	1.29 [0.59]	0.99 [0.44]	0.175

Note

Statistically significant results are indicated in bold.

Abbreviations: AP AMP, atopic asthmatic pregnant women with allergy manifestations in progeny; AP NAMP, atopic asthmatic pregnant women without allergy manifestations in progeny.

^a^ Results are presented as medians and interquartile range, median [IQR].

^b^ *p*‐values are based on the Mann‐Whitney nonparametric *U*‐test.

Women of the AP AMP group presented decreased serum IgG (*p* = 0.031) and *λ* FLC (*p* = 0.001) levels compared to AP NAMP group (Table 2). Furthermore, based on ROC curve analysis, we found that serum levels of IgG and λ FLC in pregnant women with atopic asthma were predictive factors for the manifestation of allergy in their progeny (IgG: AUC 0.79 [95% CI 0.57–1.00]; *λ* FLC: AUC 0.91 [95% CI 0.78–1.00]), allowing us to establish a cutoff of 6.74 g/L for IgG (sensitivity 90.9%, specificity 77.8%) and of 11.30 mg/L for *λ* FLC (sensitivity 81.8%, specificity 88.9%). Logistic regression analysis further supported the predictive ability of *λ* FLC (*p* = 0.023) and eventually IgG (*p* = 0.068) levels to discriminate between women in the AP group with and without allergies in their children (Table 3).

**TABLE 3:** Immune parameters associated with the development of allergy manifestations in the children of atopic asthmatic women

Parameter	OR [95% CI]^a^	*p*‐value^a^	*B* ^b^	*℮*^B^ [95% CI]^b^	*p*‐value^b^
IgG (mg/mL)	35.0 [3.1–419.5]	**0.005**	−0.654	0.520 [0.222–0.920]	0.068
λ FLC (mg/L)	36.0 [3.2–429.2]	**0.006**	−0.834	0.434 [0.166–0.749]	**0.023**

Note

Statistically significant results are indicated in bold.

B, regression coefficient; CI, confidence interval; OR, odds ratio.

^a^ Fisher's exact test.

^b^ Logistic regression analysis.

## 7 DISCUSSION

Growing evidence supports the role of B cells in maintaining maternal‐fetal tolerance and the protective equilibrium state observed during pregnancy.^9^, ^10^, ^11^ Previously, we evaluated the state of B‐cell activation in HNP, HP during the third trimester, and HP/PP by analyzing serum levels of sCD23 and BAFF.^23^ In this study, we further explored how B‐cell‐related immune mediators behave during the late pregnancy and postpartum periods in women with atopic asthma by characterizing the levels of serum sCD23, BAFF, IgG, IgM, IgA, and *κ* and *λ* FLC. To the best of our knowledge, this is the first characterization of such B‐cell activation and differentiation markers during pregnancy in women with atopic asthma.

In this study, we observed different levels of serum sCD23 levels between healthy women and those with atopic asthma. In a previous study, we found lower levels of sCD23 in women of the HP group, which increased after delivery to levels similar to those in women of the HNP group.^23^ However, in the atopic cohort, AP was associated with higher levels of sCD23 than ANP. Moreover, the levels of this marker dramatically increased during the postpartum period compared to ANP, AP, and the postpartum period of HP. Evidence indicates that levels of sCD23 are related to the differentiation of B cells from the naïve (Bm2) to the memory state.^19^ Nevertheless, the increased percentages of naïve B cells reported during pregnancy in AP^13^ were not accompanied by a decrease in levels of sCD23. Thus, in addition to the relationship with naïve/memory differentiation, sCD23 modulation in women with atopic asthma may also be driven by its function as a low‐affinity receptor for IgE. Both the receptor and its related immunoglobulin are critically linked to atopic asthma and allergic responses in both children and adults.^29^, ^30^ In fact, the release of soluble CD23 is accompanied by upregulation of IgE synthesis and secretion.^31^ Although ANP women exhibit low levels of sCD23 (probably due to the current anti‐inflammatory therapy) during pregnancy, women with atopic asthma display increases in sCD23, despite the strict control of immune responses in this period. When surpassing the pregnancy‐imposed control, CD23 rises more significantly in postpartum AP, which suggests that after delivery, the atopic condition of these women may worsen via upregulation of IgE pathways. In fact, although asthma severity usually returns to preconception levels, some authors suggest that the risk of asthma exacerbation is high immediately postpartum, with up to 46% women reporting asthma exacerbation in this period.^8^, ^32^

Unfortunately, it was not possible to assess either the levels of IgE in the studied groups (which is a limitation of the present study) or the level of asthma control during the postpartum period. To gain further insight in this context, we intend to characterize levels of IgE along the different gestational phases, including the postpartum period, and the expression levels of the surface receptor CD23 in distinct B‐cell subsets.

BAFF promotes the survival and differentiation of B cells from immature transitional B cells into a mature naïve state^33^, ^34^ and is also essential for both the implantation of the embryo and maintenance of pregnancy through secretion by the placenta.^35^, ^36^, ^37^ Accordingly, we observed increased serum BAFF levels in the third trimester in women in the AP group, as previously reported for HP,^23^, ^38^ suggesting that the atopic condition does not influence the levels of BAFF during pregnancy. Interestingly, as observed for sCD23, serum BAFF levels behaved differently in the postpartum period. Healthy women presented even higher levels of BAFF in the postpartum period compared to the third trimester of pregnancy, yet women with atopic asthma showed similar levels of BAFF in these two periods. In addition to being relevant for immature B‐cell survival and differentiation, BAFF has recently been implicated in the differentiation of some regulatory B‐cell subsets, particularly IL‐35 secreting B cells in mice.^39^ The authors indeed suggested that this new regulatory effect of BAFF might be relevant in autoimmune diseases, but it may also be important in light of our results. In fact, increased BAFF levels during pregnancy may contribute to the maintenance of a regulated environment by promoting IL‐10 secretion in B cells, as sustained by observations of both healthy pregnant women and those with atopic asthma. Multiple distinct B‐cell subsets can secrete this cytokine and they are often considered under the designation “B10 cells“, with recognized capacity to inhibit immune responses and suppress inflammation in an IL‐10 dependent way.^40^ Interestingly, the differences observed between the two postpartum groups, with higher levels of BAFF in healthy women, might result in an impaired B10 regulatory function in patients with atopic asthma. This impaired postpartum BAFF response (compared to healthy women), although present, might translate or be a cause of the underlying allergic disease, in which failure in immune regulation is known to be present. In line with this idea, we observed a positive correlation between BAFF levels and IL‐10 secreting B cells in pregnant women with atopic asthma.

Regarding immunoglobulins, we observed differences in their serum levels between healthy women and those with atopic asthma. Indeed, we found different levels of IgA between HNP, HP, and HP/PP; however, in the atopic groups, the only difference observed was an increase in IgA levels in AP/PP compared to AP. Additionally, similar to the IgG profile, the lower levels of IgA in AP increased during the postpartum period. Similar levels of IgM were observed in all groups, although healthy women showed increased postpartum levels compared to women in the HP group. A similar tendency was observed for women with atopic asthma. These data are in line with what we have previously observed for the normal evolution in B cells from late pregnancy to the postpartum period.^12^, ^14^, ^23^ If pregnancy imposes a delay in B‐cell maturation and differentiation, B‐cell function is resumed postpartum, as supported by the recovery in serum levels of all immunoglobulins (IgG, IgA, and IgM).

With regard to FLC levels, we found that the κ/λ FLC ratio increased during the postpartum period of women with atopic asthma, mainly due to the elevated levels of κ FLC levels in this period. Not surprisingly, considering that an inflammatory state is associated and required for childbirth, the augmented κ FLC levels postpartum might be a remnant of the increased activity by the adaptive immune system in the peripartum period.^41^, ^42^ In addition to the implication of light chain molecules in the pathogenesis of asthma, serum κ light chains are also reported to be increased in both atopic and nonatopic asthma, presumably reflecting a polyclonal B‐cell response.^43^ Despite the lower levels of κ FLC observed in ANP compared to HNP, this light chain was also significantly increased in women with atopic asthma in the postpartum evaluation. This observation is again in line with the reactivation of B‐cell function.

Finally, we found two possible predictive factors for allergy manifestation in the progeny of women with atopic asthma: serum levels of IgG and λ FLC. We have previously reported higher percentages of transitional B cells in women whose children presented allergy manifestations.^13^ The present study suggests that an imbalance in B‐cell‐derived markers can also have a significant impact as a risk marker. These data need to be verified in a larger cohort, though the literature already suggests a possible association between allergic diseases and levels of FLC, which can also serve as disease activity markers.^44^

This study had some limitations. First, although we managed to recruit all women at the different time points, the number of AP/PP samples used for the characterization of B‐cell activation markers was lower than those collected for the AP group. Regardless, even with the small sample size, we were able to demonstrate significant differences between the studied markers. Nevertheless, we recognize that these findings might not be applied to a broader population. Additional prospective studies should be carried out in larger cohorts with samples from additional time points, such as during the first and second trimesters of pregnancy. Despite the practical and financial difficulties in the implementation of such a large‐scale study, women should ideally be monitored prior to becoming pregnant, during pregnancy, and through the postpartum period.

In addition, as this study only included women with controlled asthma, evaluation of women with uncontrolled disease would help to clarify the relationship between B‐cell activation markers and disease course. Importantly, future research should also include local and systemic samples from the same women, as differences in the immune populations between compartments have been widely reported.^45^, ^46^ However, ethical limitations might make this assessment difficult, particularly in a pregnancy‐related setting.

Nonetheless, this study has several strengths to be highlighted. First, all clinical monitoring and laboratory tests were performed by the same team following the same protocol, and all women served as their own controls for the different pregnancy/postpartum time points evaluated. Additionally, it was possible to follow mother and child in the pursuit of relevant modifications in B‐cell markers, also associating them with the risk of allergy development in the progeny of women with atopic asthma. Nevertheless, we recognize that the number of children with allergic manifestations is likely to be an underestimation, as allergic diseases arise mostly after 6 months of age. To complete this data, we intend to continue monitoring these children till two years of age.

In conclusion, we showed that B‐cell activation markers are similarly affected by pregnancy in healthy women and in women with atopic asthma. However, after delivery, the dynamics of these parameters change significantly in the latter, particularly regarding sCD23 and BAFF serum levels. This suggests that the effects of atopic conditions are attenuated during pregnancy, ensuring a nonharmful environment for the developing fetus, but eventually start to detach from the healthy profile after birth. We also found that the levels of IgG and *λ* FLC in the serum of pregnant women with atopic asthma might be used as predictive factors for the manifestation of allergies in their children. These findings may be useful as risk markers in clinical practice for pregnant women with atopic conditions, potentially allowing the early identification and possible prevention of atopic‐related complications in their offspring.

## CONFLICT OF INTEREST

The authors declare no conflicts of interest.

## AUTHOR CONTRIBUTIONS

Study conception and design: Catarina Martins, Jorge Lima, and Luís Miguel Borrego; patient recruitment and data collection: Jorge Lima and Luís Miguel Borrego; data analysis and interpretation: Catarina Martins, Jorge Lima, Miguel Ângelo‐Dias, Geraldine Cambridge, and Maria Leandro; drafting of the manuscript: Catarina Martins, Miguel Ângelo‐Dias, and Jorge Lima; critical revision of the manuscript: Geraldine Cambridge, Maria Leandro, and Luís Miguel Borrego. All authors approved the final version of the manuscript and take responsibility for the accuracy and integrity of any part of the work.

## Supporting information

Figure S1

## Data Availability

The data that support the findings of this study are available from the corresponding author upon reasonable request.
