# Supplementary figures and images for: Serum markers of B‐cell activation in pregnant women with atopic asthma
Source: Am J Reprod Immunol. 2021 Mar 19;86(2):e13414. doi: 10.1111/aji.13414 (PMC8365761; doi:10.1111/aji.13414)

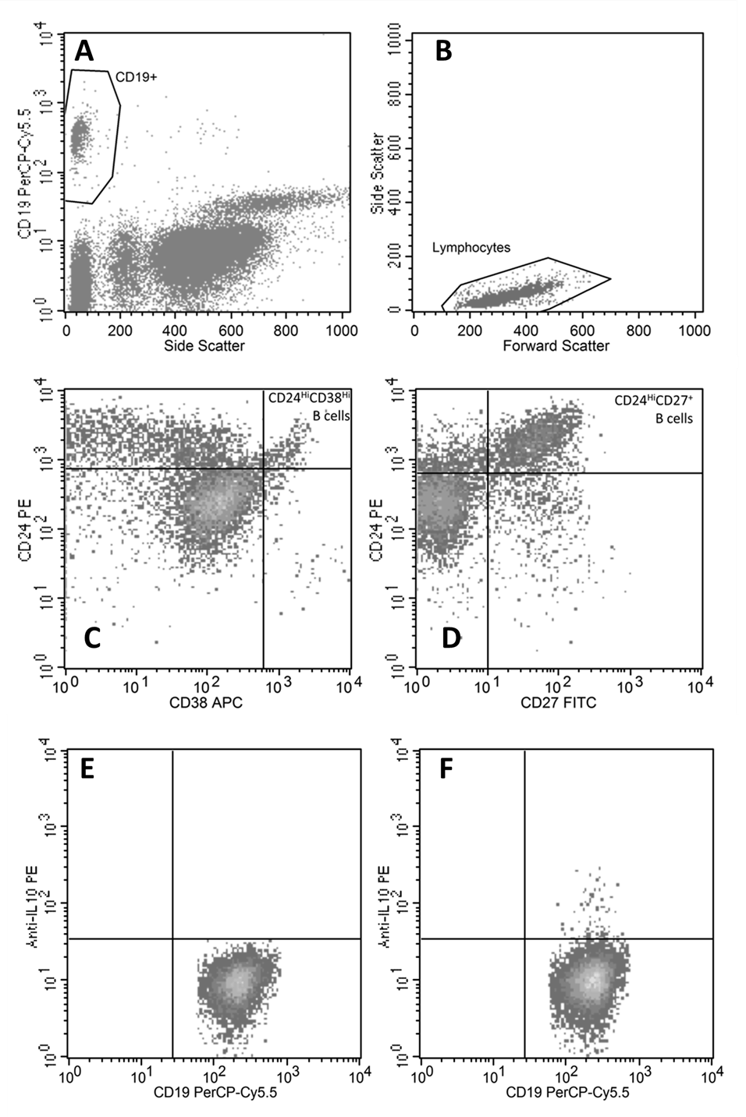

Supplement: Supplementary file 1 — Figure S1 [file AJI-86-e13414-s001.tif]
